
JOURNAL INFORMATION
==============================
NLM Title Abbreviation: ScientificWorldJournal
Journal ID: ScientificWorldJournal
NLM Journal ID: 101131163
Journal ID: TSWJ

The Scientific World Journal

ISSN: 2356-6140
EISSN: 1537-744X
Publisher: Wiley

ARTICLE INFORMATION
==============================
PMCID: PMC5901321
PMID: 29861696
DOI: 10.1100/tsw.2007.198
Article ID: 431386
Article version: 1
Subjects: Editorial

Eicosanoid Receptors and Their Role in Regulating Immune and Inflammatory Responses

Brink Charles
CNRS CR-1, Paris, France
Publication date: 2007
Electronic publication date: 2007 Sep 1
Volume: 7
First page: 1272
Last page: 1272
Copyright: Copyright © 2007 Charles Brink.
Copyright year: 2007
License: This is an open access article distributed under the Creative Commons Attribution License, which permits unrestricted use, distribution, and reproduction in any medium, provided the original work is properly cited.
License URL: https://creativecommons.org/licenses/by/3.0/

==============================
Editorial
Special Issue: Eicosanoid Receptors and Inflammation
*TheScientificWorldJOURNAL* (2007) 7, 1272
ISSN 1537-744X; DOI 10.1100/tsw.2007.198

TheScientificWorldJOURNAL
www.thescientificworld.com

### Eicosanoid Receptors and Their Role in Regulating Immune and Inflammatory Responses

Charles Brink
*CNRS CR-1 PARIS, France*

**E-mail**: charlesbrink@hotmail.com

*Received July 9, 2007; Accepted July 9, 2007; Published September 1, 2007*

---

There is an array of lipid mediators produced from arachidonic acid. These metabolic products derived from membrane phospholipids, by the actions of phospholipases, act by signaling through G-protein coupled receptors. To better appreciate their impact and role during inflammation and the immune response, this issue of the *TheScientificWorldJOURNAL* brings into perspective our current biochemical knowledge of the eicosanoids, as well as information on their receptors and signaling pathways.

Since the hallmark of inflammation is a major perturbation in the vasculature, a considerable amount of information is reviewed dealing with blood cells and the vascular wall, as well as eicosanoid receptors and cellular signals observed in different animal models and human tissues. Although metabolites of the arachidonic acid cascade have often been documented to facilitate and enhance the inflammatory response, there are several metabolites that may markedly alter the intensity and duration of the reaction, thereby dampening this complex response. A considerable interest has arisen, therefore, to explore not only the proinflammatory, but also anti-inflammatory, eicosanoid actions during different phases of the inflammatory reaction.

While the eicosanoids and their receptors cooperate with many other molecules during the various phases of the inflammatory reaction, an attempt has been made to highlight the capacity of the eicosanoids to signal in the immune system by addressing their role in the "cross-talk" between disparate cell types during the immune response.

The papers presented in this issue hopefully will provide the reader with a summary of the eicosanoid receptor diversity and their association with both inflammation and the immune response.

---

**This article should be cited as follows:**

Brink, C. (2007) Eicosanoid receptors and their role in regulating immune and inflammatory responses. *TheScientificWorldJOURNAL* **7**, 1272. DOI 10.1100/tsw.2007.198.

---

©2007 with author.
Published by TheScientificWorld; www.thescientificworld.com
